# Challenges in assessing self-reported adherence to antiretroviral therapy among individuals living with HIV/AIDS and mental disorders

**DOI:** 10.1186/s13104-025-07368-z

**Published:** 2025-07-16

**Authors:** Camila Guadeluppe Maciel, Priscilla Arashiro, Lívia Alves da Silva, Ana Isabel do Nascimento, Danilo dos Santos Conrado, Gabriel Serrano Ramires Koch, João Cesar Pereira da Cunha, Laysa Gomes Osório, Letícia Suemi Arakaki, Lisany Krug Mareto, Maria Eduarda de Souza Rodrigues, Micael Viana de Azevedo, Robson França Gomes e Silva, Rodrigo Mayer Pucci, Samara Tessari Pires, Sara Raquel Pinto Borges, Simony Portela do Carmo Drumond, Márcio José de Medeiros, Maria Elizabeth Araújo Ajalla, Cláudia Du Bocage Santos-Pinto, Everton Falcão de Oliveira

**Affiliations:** 1 Secretaria Municipal de Saúde Pública de Campo Grande, Campo Grande, MS Brasil; 2 Programa de Pós-Graduação em Doenças Infecciosas e Parasitárias, Universidade Federal de Mato Grosso do Sul, Campo Grande, MS Brasil; 3 Hospital Universitário Maria Aparecida Pedrossian, Universidade Federal de Mato Grosso do Sul, Campo Grande, MS Brasil; 4 Faculdade de Medicina, Universidade Federal de Mato Grosso do Sul, Campo Grande, MS Brasil; 5 Instituto Politécnico, Universidade Federal do Rio de Janeiro, Macaé, RJ Brasil

**Keywords:** Self-reported adherence, Antiretroviral therapy, Mental disorders, Substance use-related mental disorders

## Abstract

Studies focusing on individuals living with HIV and mental disorders are crucial to inform and enhance care for this inherently vulnerable population group, especially considering that people living with HIV/AIDS (PLHIV) are more likely to develop mental disorders compared to the general population, contributing to lower adherence to antiretroviral therapy (ART). This study cross-sectional study aimed to assess self-reported adherence to antiretroviral therapy among PLHIV and moderate or severe mental disorders in Campo Grande, Mato Grosso do Sul, Brazil. Patient-reported data from PLHIV who received care in at least one of the Psychosocial Care Network facilities of the Brazilian Unified Health System from 2014 to 2018 were collected to assess the adherence to ART. Among the 76 participants eligible, 35 were included in the study. Substance use-related mental disorders were the most prevalent (45.7%), followed by mood disorders (25.7%) and anxiety (11.4%). Most of the participants had a low ART adherence (62.9%), followed by insufficient adherence (22.9%) and good adherence (14.3%). No significant associations were found between the adherence to ART and the study variables. Our findings suggest the importance of assessing adherence based on both direct and indirect measures, as biological markers and self-report.

## Introduction

More than 40 years after the onset of the HIV/AIDS epidemic, several challenges persist in controlling and reducing the estimated number of new cases—around 1.3 million new infections worldwide in 2022 [1]. There is a need to expand access to early diagnosis, treatment, and ongoing care, particularly in vulnerable populations and key groups [2, 3]. Despite the reduction in new HIV infections and AIDS-related deaths over the last decade, HIV/AIDS is projected to become a global endemic by 2030 [2]. 

As the transition from epidemic to endemic progresses, various health needs emerge. During an epidemic, the proposed control and management strategies are predominantly collective, involving the prioritization of resources for prevention and treatment to reduce the risk of HIV infection [4]. In contrast, the endemic scenario necessitates responses tailored to the individual context and the management of a chronic health condition. This includes addressing changes in lifestyle habits and ensuring adherence to antiretroviral therapy (ART) for diverse population profiles, given that the disease affects individuals across all age groups and social classes [2, 4]. 

Adherence to ART and, consequently, the success of drug treatment is influenced by various factors, both individual and systemic. Individual factors include socioeconomic conditions, while health system factors encompass access to health services and medication [5–7]. Notably, among the individual factors, the well-established correlation between low adherence or abandonment of ART and mental disorders stands out, especially considering that people living with HIV/AIDS (PLHIV) are more likely to develop mental disorders compared to the general population, contributing to lower adherence to ART [8–10]. 

Among the factors associated with mental disorders, substance use, anxiety, hopelessness, feelings of guilt, and cognitive disorders affecting attention and memory are closely linked to low therapeutic adherence [6, 7, 11]. A compounding factor, often the catalyst for these disorders, is the social stigma attached to PLHIV. This stigma can result in discrimination and prejudice, directly impacting outpatient follow-up and medication adherence [6, 7, 12]. Additionally, it’s crucial to consider that, due to the COVID-19 pandemic, there has been an increased occurrence of anxious and depressive mental disorders [3]which may have a direct impact on the medication adherence of PLHIV.

In this context, studies focusing on individuals living with HIV and mental disorders are crucial to inform and enhance care for this inherently vulnerable population group. The various psychosocial challenges they encounter can create an additional burden, compromising both their overall health condition and the maintenance of good adherence to treatment.

Self-reported adherence is particularly relevant in populations with mental disorders, where traditional measures such as pill counts and pharmacy refill records may be less reliable due to irregular healthcare utilization and complex medication-taking behaviors [13]. Additionally, self-report methods may be more feasible in these contexts, allowing for the assessment of both intentional and unintentional non-adherence, particularly when structured and validated instruments are used [14]. 

Therefore, the study aimed to assess self-reported adherence to antiretroviral therapy among people living with HIV/AIDS and mental disorders in Campo Grande, Mato Grosso do Sul, Brazil.

## Methods

### Study design, setting, and data source

This cross-sectional study used self-reported data from PLHIV that was collected based on the application of the updated version of the *Cuestionario para la Evaluación de la Adhesión al Tratamiento Antiretroviral* (CEAT-VIH) [13, 14] patient-reported outcome measure, which assesses five dimensions of adherence: treatment compliance, antecedent non-adherence behaviors, doctor-patient communication, treatment expectations, and treatment satisfaction.

Individuals living with HIV aged over 18, who received care in at least one of the Psychosocial Care Network (PCN) facilities of the Brazilian Unified Health System in Campo Grande from 2014 to 2018, were considered eligible for this study. During this period, the PCN in Campo Grande had five Psychosocial Care Centers, which offer open and community-based health services to people with moderate and severe mental disorders, including those with needs related to alcohol and drug abuse, in addition to the Street Clinic teams that provide care to the homeless population.

The manual record-linkage was used to cross-reference data from two databases and identify individuals eligible for the study: National System of Disease Notification (SINAN) and the Outpatient Information System (SIA-SUS). SINAN contains cases of notifiable diseases and SIA-SUS contains records of outpatient and specialized care, including care provided at Psychosocial Care Centers visits. The search period on SINAN was from 1984, when AIDS reporting began in Brazil, to 2018; on SIA-SUS it was between 2014 and 2018. Names, national health card numbers, date of birth, and mother’s name used for the manual record linkage to identify individuals eligible to study.

After identifying the individuals eligible for the study, they were contacted and invited to participate. Following the acquisition of informed consent, interviews were conducted to administer the CEAT-HIV. Initially planned for in-person sessions at participants’ outpatient clinics, the interviews shifted to a telephone format between May and July 2021 due to the COVID-19 pandemic. To minimize potential biases, questions were read slowly and clearly, and participants were encouraged to request clarification when necessary. For homeless people, a member of the specialized Street Clinic teams administered the CEAT-HIV.

Data on individuals’ clinical–epidemiological profiles, including the diagnoses, were collected from the HIV/AIDS notification forms in SINAN, medical records held at the PCN care points, and centers specializing in infectious and parasitic diseases. The electronic medical records available in the Information Management System of the Municipal Health Department and the Street Clinic service reports were also reviewed. This system is specific to the place where the study was conducted and includes data such as patient reports on living conditions (e.g., homelessness), substance use, suicidal ideation, and the frequency of attendance at specialized outpatient clinics.

Data regarding the ART dispensing history and the number individuals living with HIV who were on antiretroviral treatment during the study period were extracted from the Brazilian Medication Logistics Control System – SICLOM. HIV viral load and CD4 + cell counts were obtained from the Laboratory Test Control System of the National Counting Network for CD4+/CD8 + Lymphocytes and HIV Viral Load – SISCEL and refer to the closest count to the date of the interview and application of the CEAT-VIH.

### Outcome measure and definitions

Adherence to ART was assessed based on the CEAT-VIH adherence raw score, that range from 17 to 85 points, being: low adherence – less than or equal to 74; insufficient adherence – between 75 and 79; good adherence – greater than or equal to 80 [15]. 

Mental disorders were categorized as described by Arashiro et al., [17] which consists of the mental disorders into four categories according to the chapters of the ICD-10: anxiety (F40-F48), mood/depression (F30-F39), substance use (F10-F19), and other disorders, which grouped disorders not included in the previous categories.

Other clinical–epidemiological data assessed were: time of HIV infection (years); possible mode of transmission of HIV; sexual behavior/practice; co-infections; drug use; alcohol use; suicidal ideation; having regular follow-up at Psychosocial Care Centers: at least one appointment with a psychiatric doctor every 2 months, in addition to consultations or home visits conducted by the multidisciplinary team as part of the singular therapeutic project; and having regular follow-up at services specialized in infectious and parasitic diseases: at least one appointment with an infectious disease doctor every 6 months.

Sociodemographic data included were sex, age, race/skin color, educational level, and history of homelessness.

### Data analysis

The qualitative variables were described using the measures number of occurrences ($\:n$) and proportions (%), the data were presented in frequency distributions. For continuous variables, the descriptive measures were mean, median, and standard deviation.

The t-test for paired data was used to assess the differences of means of viral load and CD4 cells between the beginning and end of period of follow up.

The association between adherence to ART and the epidemiological and clinical data was assessed by an univariable analysis. At this point, the adherence score was categorized into good and low (which grouped the categories low and insufficient) adherence to ART. The Mann-Whitney *U* test was used for continuous variables and Fisher’s exact test for categorical variables. The significance level adopted for all hypothesis tests was 5% (α = 0.05). The analysis was performed using R software version 4.0.4, and the following packages were used: *tidyverse* and *descr*.

## Results

**Fig. 1:**
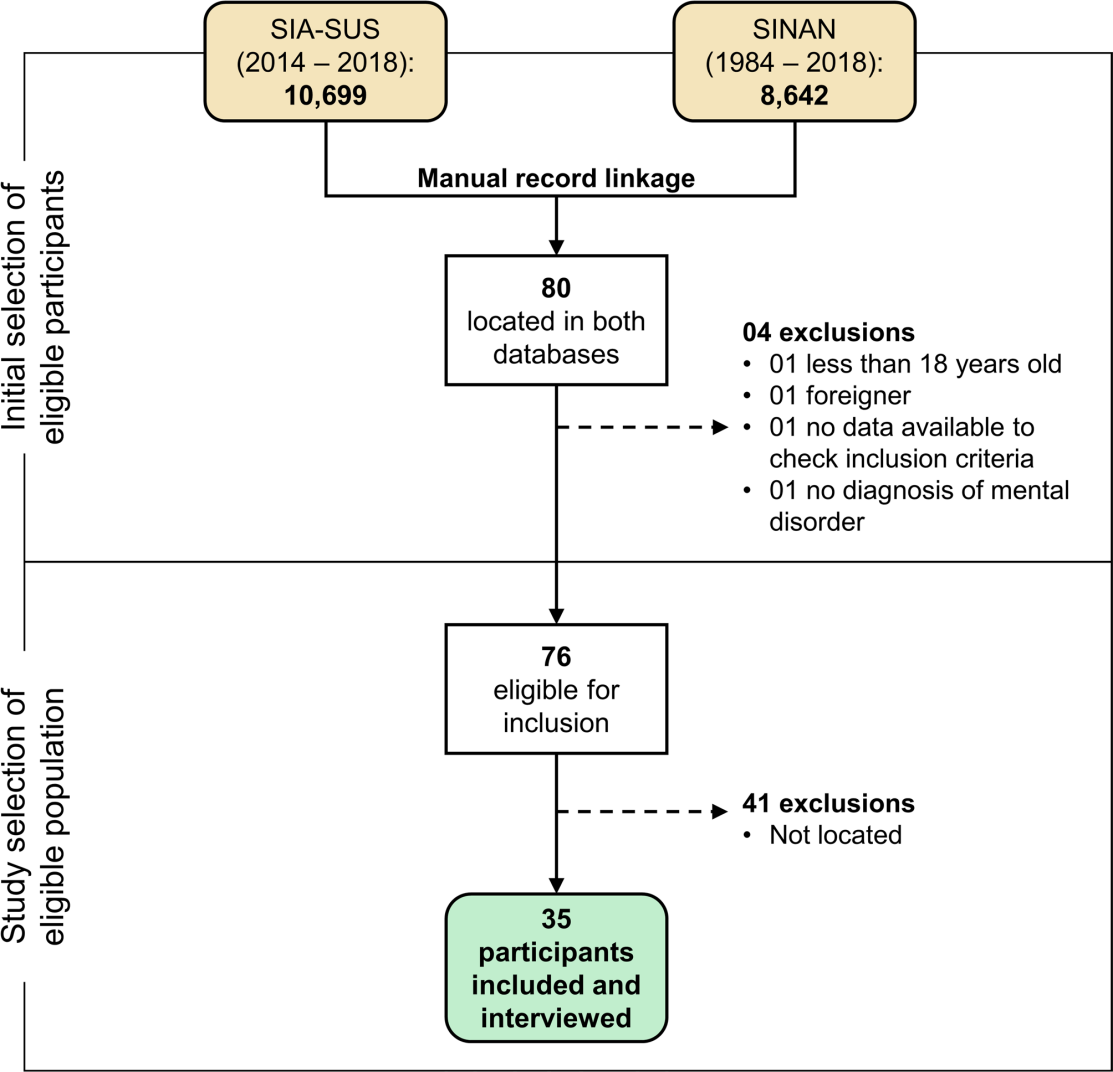
Flowchart of identification, eligibility assessment, and recruitment of study participants

Figure 1 illustrates the procedure for locating individuals in the study. Following the manual record linkage, 80 individuals were simultaneously identified in SINAN and SIA-SUS. Among them, four individuals were excluded from the study: one under 18 years old, one foreigner with records limited to HIV/AIDS notification and care at PCN facilities, one due to the absence of reports or necessary data needed for the study, and one patient deemed ineligible for lacking a moderate or severe disorder despite a history of care at a Psychosocial Care Center. Consequently, 76 participants were eligible for the study, but only 35 were successfully contacted by telephone for the interview and administration of the CEAT-HIV.

The epidemiological and clinical profile of these 35 participants is summarized in Table 1.

**Table 1:** Epidemiological and clinical characteristics of the study population ($\:n$ = 35)

Variable	$\:\varvec{n}$	%
**Age (year) on data collection**		
18 to 30	15	42.9
31 to 40	11	31.4
41 to 60	9	25.7
**Age (year) at the time of the HIV/AIDS diagnosis report**		
18 to 30	10	28.6
31 to 40	10	28.6
41 to 60	13	37.1
61 and more	2	5.7
**Time living with HIV (from diagnosis to data collection)**		
Up to 5 years	12	34.3
More than 5 years	23	65.7
**Sex**		
Female	12	34.3
Male	23	65.7
**Race or skin color**		
White	16	45.7
Non-white	17	48.6
Ignored	2	5.7
**Educational level**		
Elementary school (1 to 9 years of study)	11	31.4
High school (10 to 12 years of study)	1	2.9
Higher education (over 13 years of study)	2	5.7
Ignored	21	60.0
**Homeless**		
No	29	82.9
Yes	6	17.1
**Mental disorder**		
Anxiety	4	11.4
Mood	9	25.7
Related to substance use	16	45.7
Others	6	17.1
**Abuse of alcohol**		
No	22	62.9
Yes	13	37.1
**Injection drug use**		
No	21	60.0
Yes	14	40.0
Ignored		
**Suicidal ideation**		
No	21	60.0
Yes	14	40.0
**Regular follow-up at Psychosocial Care Centers**		
No	19	54.3
Yes	16	45.7
**Co-infections**		
No	22	62.9
Yes	13	37.1
**Viral load**		
Not detected or less than 40	28	80.0
Detectable	6	17.1
Data not available	1	2.9
**CD4 + cells**		
Less than 200	5	14.3
201 to 350	1	2.9
350 to 500	4	11.4
Higher than 500	24	68.6
Data not available	1	2.9
**Regular follow-up in the specialized outpatient infectious disease facility**		
No	15	42.9
Yes	18	51.4
Data not available	2	5.7

Data source: Notifiable Diseases Information System (SINAN), Medication Logistics Control System (SICLOM) and data obtained from the review of outpatient and hospital records of follow-up services in 2021

Most participants were between 18 and 30 years old at the time of the interview (42.9%), with a majority being men (65.7%) and non-white individuals (48.6%). The majority had been diagnosed with HIV/AIDS for more than 5 years at the time of data collection. Substance use-related mental disorders were the most prevalent (45.7%), followed by mood disorders (25.7%) and anxiety (11.4%). Less than half of the participants reported regular attendance at Psychosocial Care Center (45.7%), while 40.0% reported suicidal ideation. Regarding viral load, 80.0% had undetectable or less than 40 copies/mm³, and 68.6% had a CD4 cell count greater than 500 cells/mm³ (Table 1). Just over half (51.4%) were regularly followed up in HIV/AIDS treatment referral services.

Based on the application of the CEAT-VIH, most of the participants had a low degree of adherence (62.9%), followed by insufficient adherence (22.9%) and good adherence (14.3%).

**Fig. 2:**
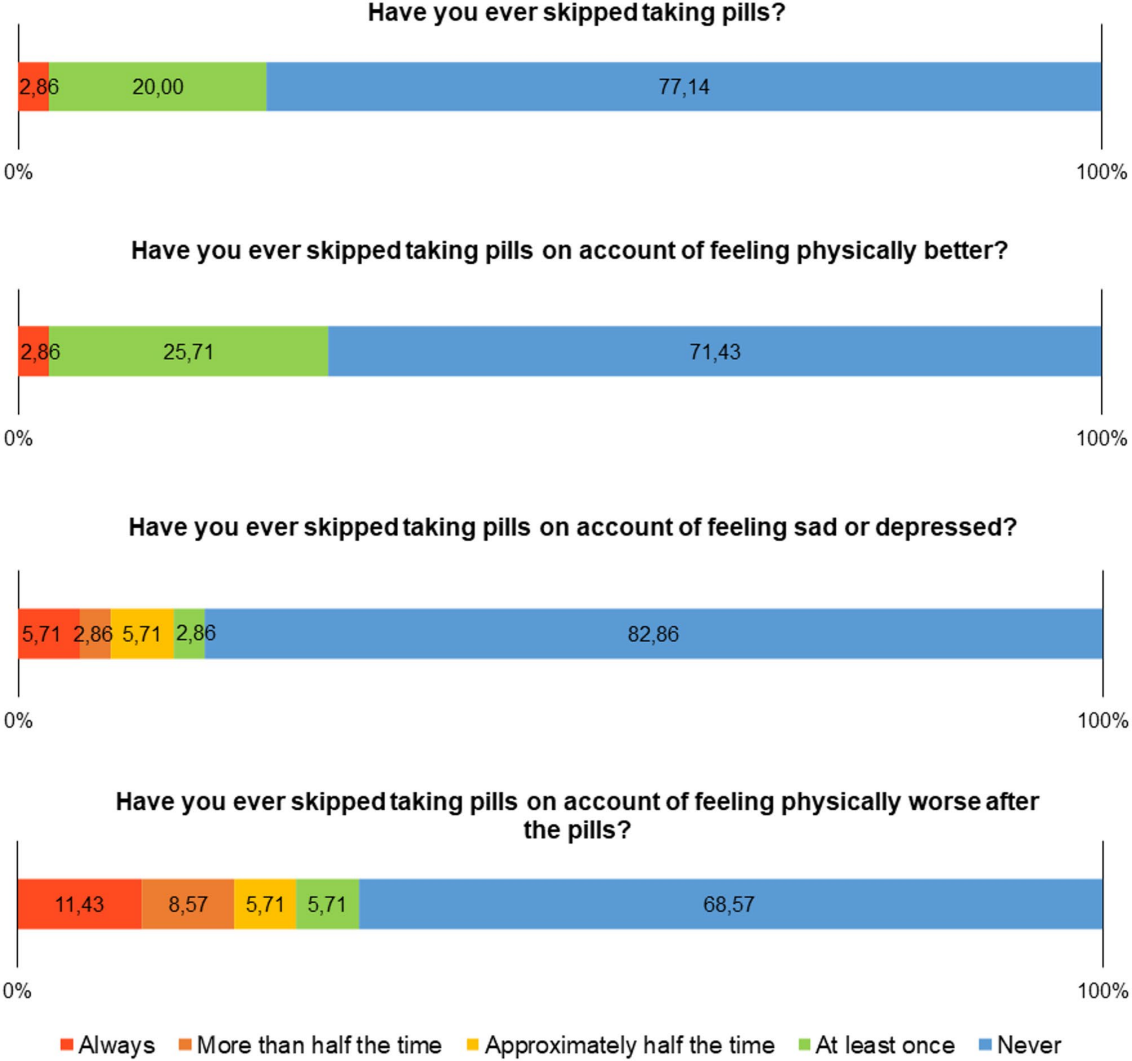
Frequency distribution of factors related to non-adherence to ART

Figure 2 shows the details of the findings for the questions on the dimension antecedents of non-adherence, in which it can be seen that the majority of participants did not report not taking their medication in the 30 days prior to the date of the interview.

**Fig. 3:**
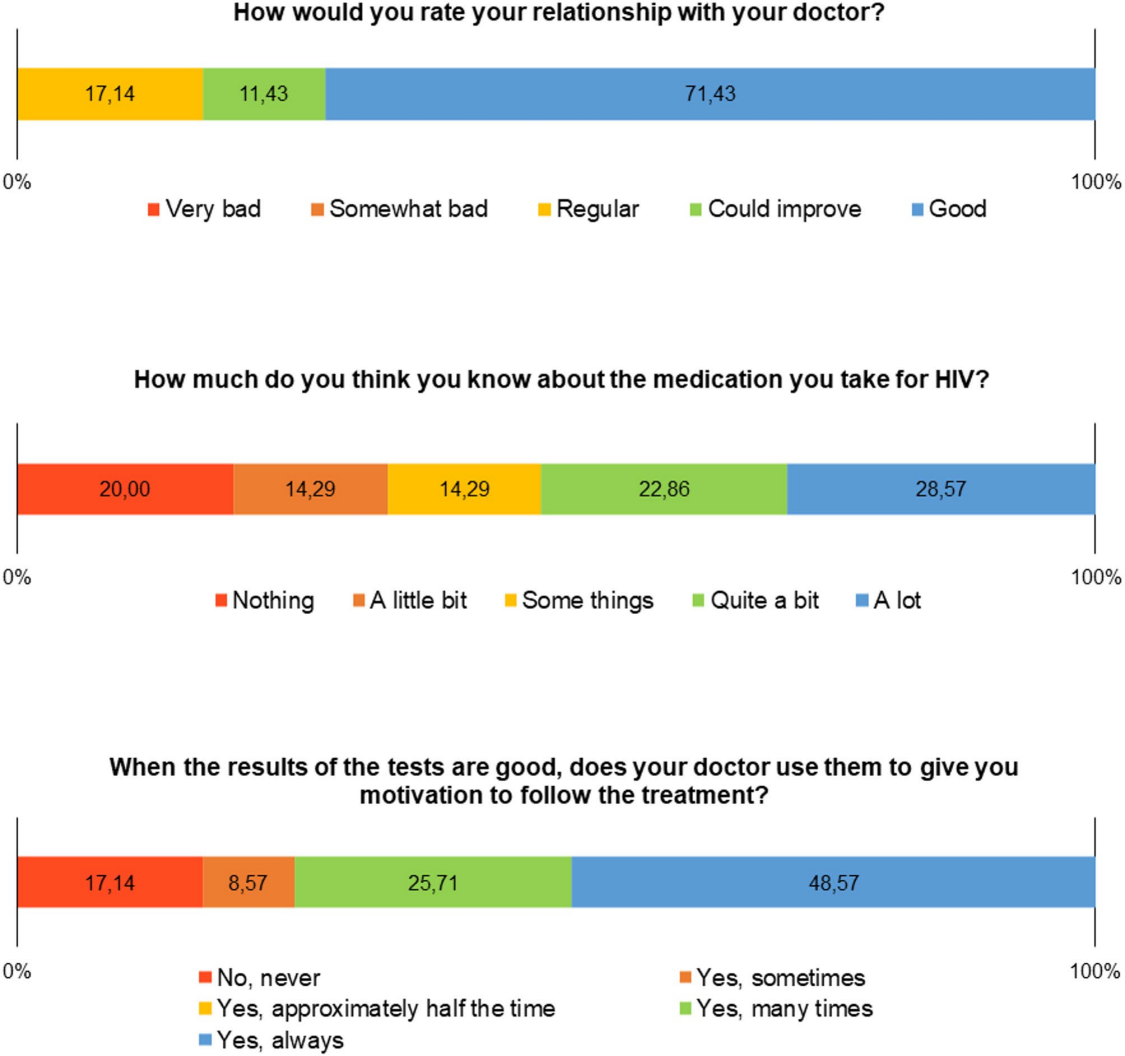
Frequency distribution of doctor-patient relationship scores domain of the CEAT-HIV scale

When assessing the doctor-patient relationship, although almost three-quarters of the participants consider this relationship to be good, it is noteworthy that almost half do not know the necessary information about ART, which can include guidelines and strategies for use, dosages, adverse effects, drug interactions, among others (Fig. 3).

**Fig. 4:**
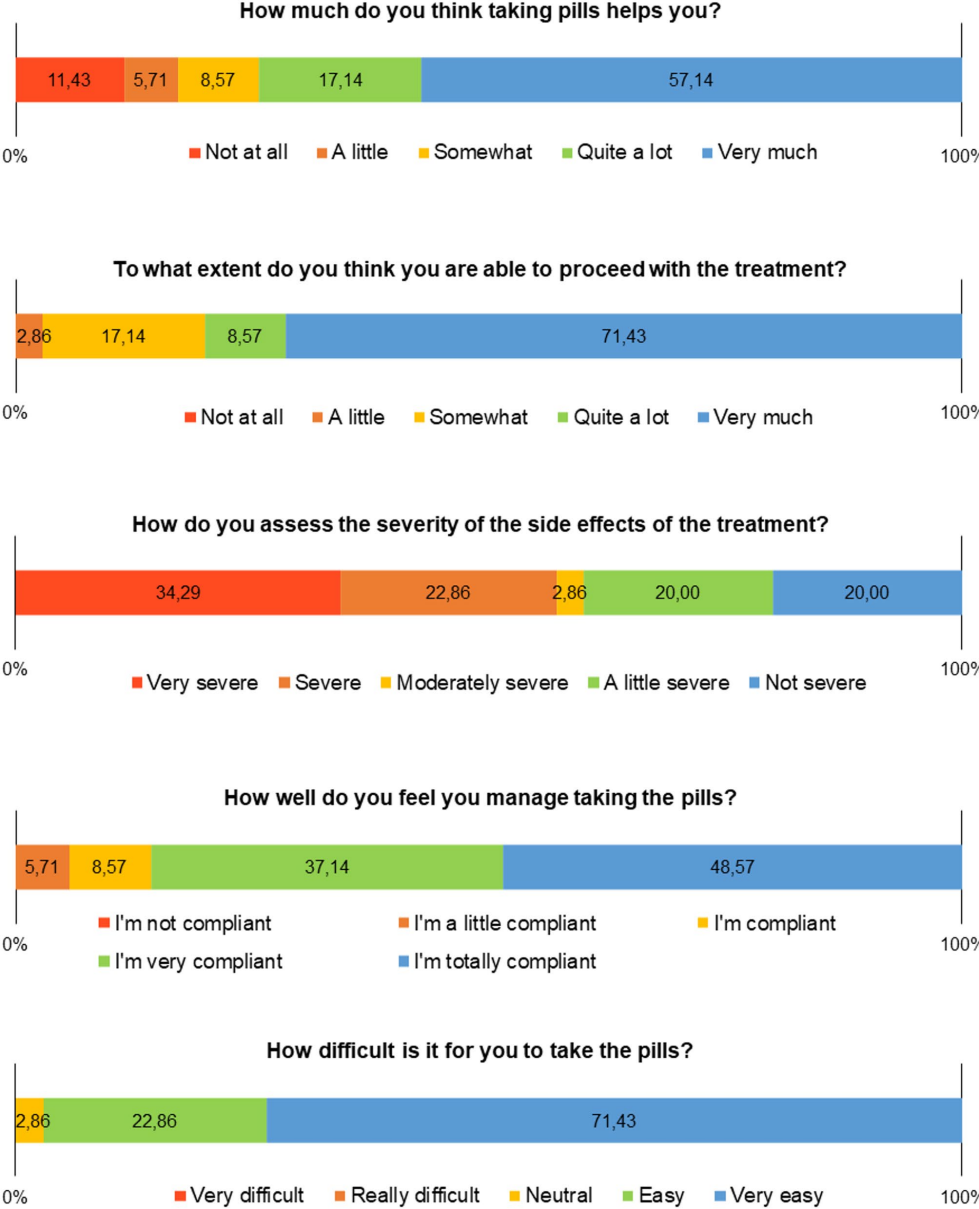
Frequency distribution of beliefs and expectations regarding ART

With regard to beliefs and expectations about treatment, there was a high percentage of participants reporting moderate and high adverse effects (57.0%), which could be a negative indicator of the frequency and intensity of adverse effects in relation to drug treatment, contributing significantly to low adherence. However, the majority said that they felt that ART was beneficial and that they felt able to continue with the treatment (Fig. 4).

**Fig. 5:**
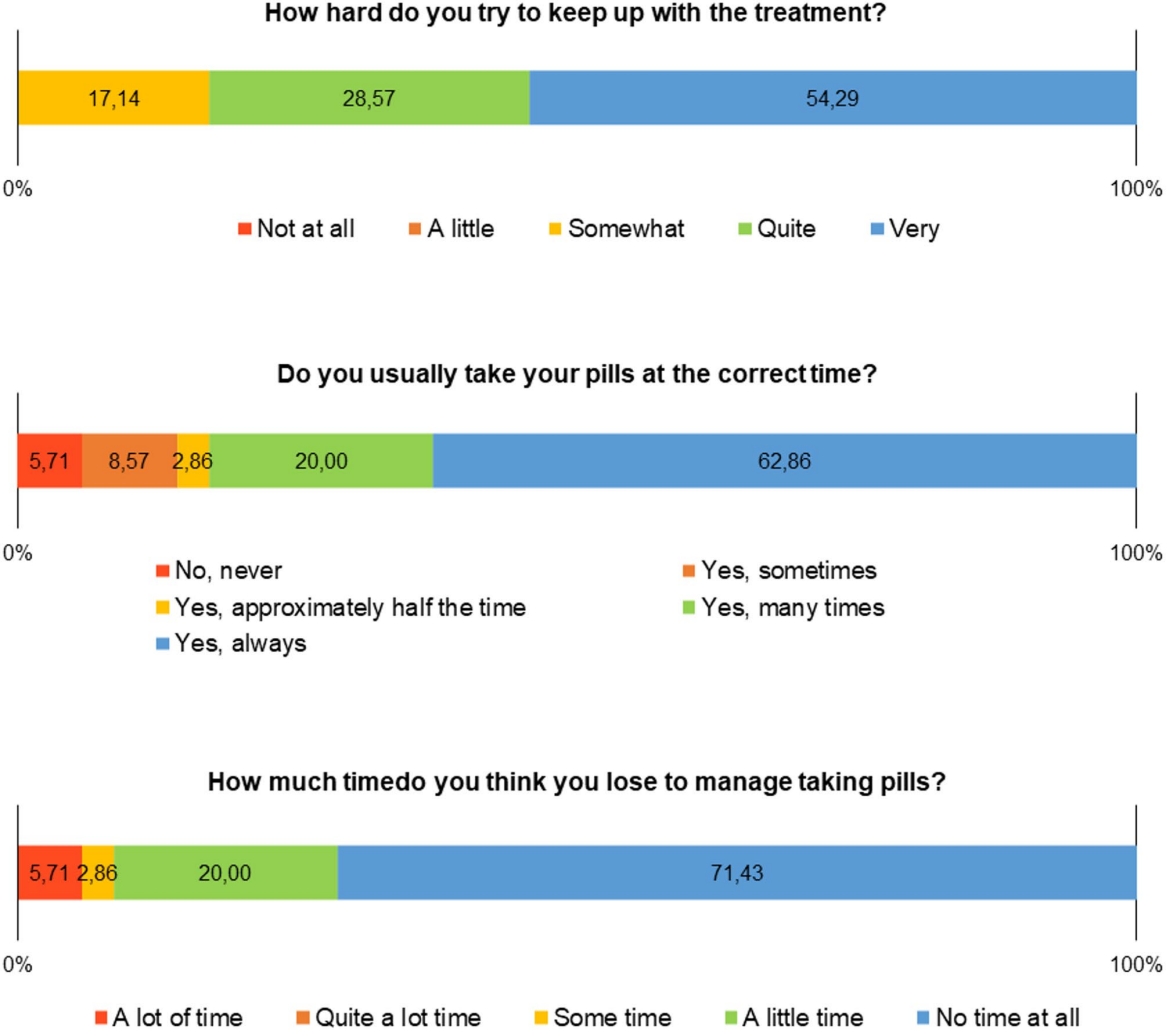
Frequency distribution of responses in the CEAT-HIV scale domain related to compliance with ART

In the set of questions on compliance with treatment, positive findings are highlighted in relation to following the health team’s instructions on taking the medication, including taking the medication at a fixed time (Fig. 5).

**Fig. 6:**
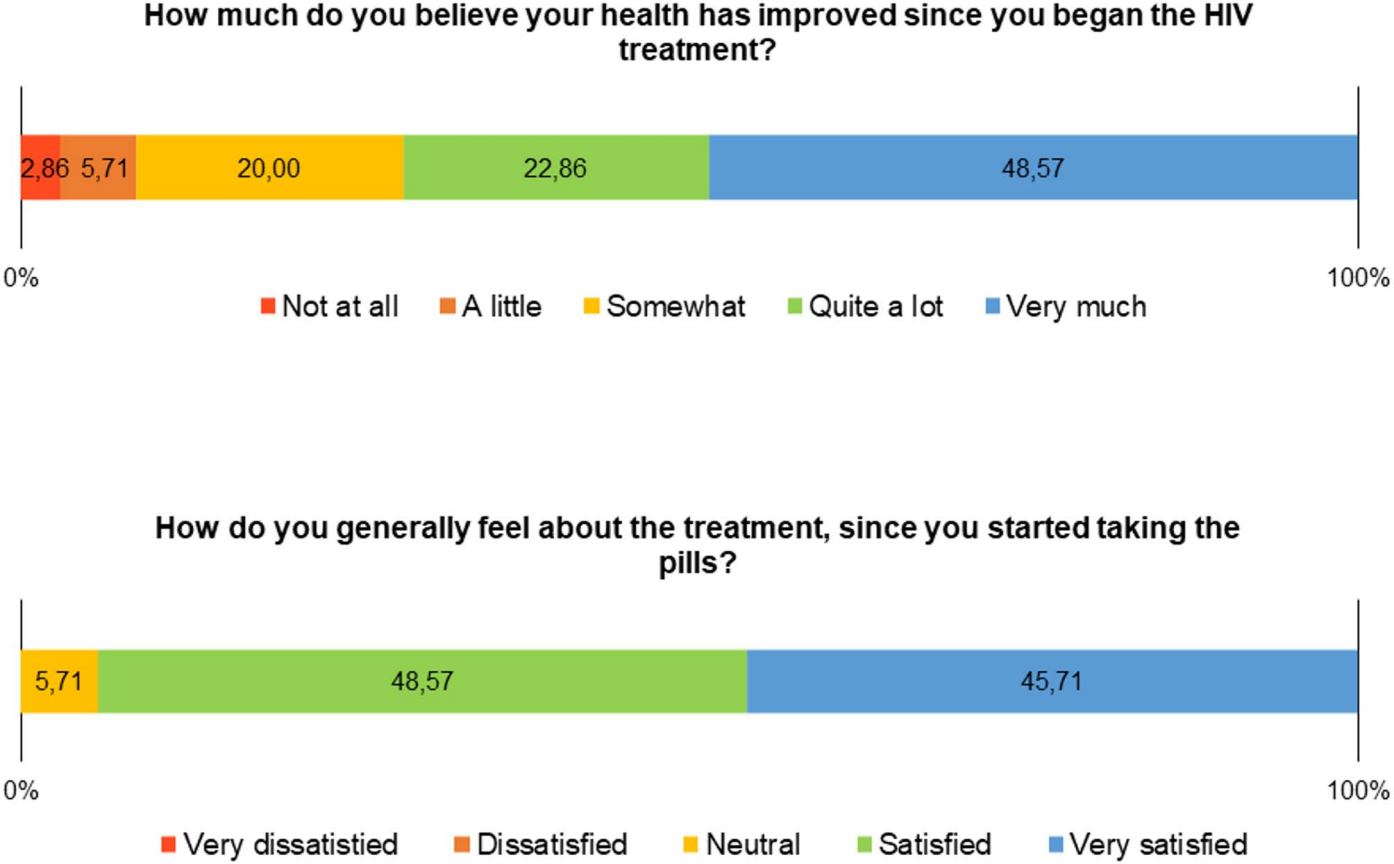
Frequency distribution of responses in the satisfaction with treatment domain of the CEAT-HIV scale

The two questions that express the dimension of satisfaction with treatment show good satisfaction and perception of health status after starting ART (Fig. 6), even in the face of the side effects mentioned in Fig. 4.

In the analysis of association (Table 2), no significant associations were found between the raw score of the CEAT-HIV for adherence to ART and the other study variables.

**Table 2:** Association between adherence to ART and clinical-epidemiological variables ($\:n$ = 35)

Continuous variables	Adherence		*p*-value*
	Good	Low	
	Mean (SD)	Mean (SD)	
**Age (year) on data collection**	44.0 (10.4)	38.9 (12.2)	0.388
**Age (year) at the time of the HIV/AIDS diagnosis report**	34.2 (11.3)	32.9 (10.0)	0.794
**Time living with HIV (from diagnosis to data collection)**	10.6 (8.6)	7.3 (5.4)	0.252
**Categorical variables**	$\:\varvec{n}$ **(%)**	$\:\varvec{n}$ **(%)**	
**Sex**			
Female	0 (0.0)	12 (100.0)	0.141
Male	5 (21.7)	18 (78.3)	
**Race or skin color**			
White	2 (12.5)	14 (87.5)	1.000
Non-white	3 (17.6)	14 (82.4)	
**Educational level**			
Elementary school (1 to 9 years of study)	1 (9.1)	10 (90.9)	0.154
High school (10 to 12 years of study)	1 (100.0)	0 (0.0)	
Higher education (over 13 years of study)	0	2 (100.0)	
**Homeless**			
No	5 (17.2)	24 (82.8)	0.561
Yes	0 (0.0)	6 (100.0)	
**Mental disorder**			
Anxiety	1 (25.0)	3 (75.0)	0.427
Mood	0 (0.0)	9 (100.0)	
Related to substance use	3 (18.8)	13 (81.2)	
Others	1 (16.7)	5 (83.3)	
**Abuse of alcohol**			
No	4 (18.2)	18 (81.8)	0.630
Yes	1 (7.7)	12 (92.3)	
**Injection drug use**			
No	2 (9.5)	19 (90.5)	0.369
Yes	3 (21.4)	11 (78.6)	
**Suicidal ideation**			
No	4 (19.0)	17 (81.0)	0.627
Yes	1 (7.1)	13 (92.9)	
**Regular follow-up at Psychosocial Care Centers**			
No	1 (5.3)	18 (94.7)	0.156
Yes	4 (25.0)	12 (75.0)	
**Co-infections**			
No	2 (9.1)	20 (90.9)	0.337
Yes	3 (23.1)	10 (76.9)	
**Viral load**			
Not detected or less than 40	5 (17.9)	23 (82.1)	0.559
Detectable	0 (0.0)	6 (100.)	
**CD4 + cells**			
Less than 200	1 (20.0)	4 (80.0)	0.127
201 to 350	0 (0.0)	1 (100.0)	
350 to 500	2 (50.0)	2 (50.0)	
Higher than 500	2 (8.3)	22 (91.7)	
**Regular follow-up in the specialized outpatient infectious disease facility**			
No	1 (6.7)	14 (93.3)	0.346
Yes	4 (22.2)	14 (77.8)	

Data source: Notifiable Diseases Information System (SINAN), Medication Logistics Control System (SICLOM) and data obtained from the review of outpatient and hospital records of follow-up services in 2021

*Fisher’s exact test

SD: Standard Deviation

## Discussion

The prevalence of mental disorders associated with HIV is estimated to range between 18% and 50% [18]. Despite this considerable prevalence and the growing number of studies aimed at understanding the pathophysiology of HIV-associated mental disorders, there is currently no definitive marker or specific treatment for preventing these issues in PLHIV [19]. Hence, research focusing on advancements in antiretroviral drugs, the promotion of social awareness to reduce stigma, and the investigation of factors affecting adherence to ART is crucial for enhancing the prognosis of PLHIV. Currently, ART remains the sole option to prevent or delay the progression of HIV-associated mental disorders, though its effectiveness is limited to a subset of patients [18–21]. This study, assessing self-reported adherence to ART among PLHIV with mental disorders in the psychosocial care network, identifies potential intervention points, such as inadequate drug information and the high intensity of side effects, that may compromise ART adherence.

Our study population comprised predominantly young adult men, non-white, with limited education, diagnosed with HIV/AIDS for over five years, and maintaining an undetectable viral load. This profile closely aligns with the overall scenario of PLHIV in Brazil [22, 23], underscoring the necessity for heightened attention to young individuals. Emphasis should be placed on preventive measures, including enhanced support, guidance on the utilization of pre- and post-exposure prophylaxis for HIV risk behaviors. Additionally, addressing the risks linked to substance abuse is crucial, given the frequent occurrence of mental disorders associated with drug use in our analysis and other studies. These findings support the correlation between drug use and the emergence of depressive symptoms [24, 25], compromised adherence to ART, heightened susceptibility to opportunistic diseases, and increased mortality risk [26]. 

The elevated prevalence of individuals with a history of suicidal ideation, both in our study and in other research involving PLHIV [27]underscores the critical need to advocate for comprehensive psychological support interventions for HIV patients. This is particularly crucial given the negative correlation between psychopathological symptoms such as anxiety, depression, and suicidal ideation, and adherence to treatment [28, 29]. Implementing comprehensive care and follow-up strategies, involving multi-professional teams and incorporating non-pharmacological measures, becomes imperative for the successful adherence to ART and the control and prevention of HIV-related mental disorders [18, 30]. 

Our findings are consistent with previous studies reporting challenges in achieving optimal adherence in individuals with mental disorders where cognitive impairments, symptoms of psychiatric conditions, and stigma were found to contribute to poor adherence among individuals with severe mental illness [11, 17, 26]. Adherence to ART is a multifaceted and dynamic process, influenced by several factors such as age, education level, and the strength of connections with healthcare teams within the patient’s care network [17, 31–33]. Our results did not reveal significant associations between adherence to antiretroviral therapy and the study variables. However, given the small sample size, the study may have been underpowered to detect statistically significant differences. Additionally, unmeasured factors, such as social support and perceived stigma, may play a more substantial role in influencing adherence among individuals living with HIV. Previous studies have demonstrated that greater social support is associated with higher levels of medication adherence [34]while HIV-related stigma has been identified as a significant barrier to adherence [6, 7]. 

The complexity of adherence poses challenges in measurement using a singular scale or across various methods, encompassing indirect measures like self-reporting [15, 16, 33], drug dispensing monitoring, and pill counting [32, 33], as well as direct measures such as detecting antiretroviral drugs in the blood, viral load, and CD4 + cell counts [35]. 

Our study, examining self-reported ART adherence among PLHIV with moderate or severe mental disorders, predominantly classified the majority of patients as non-adherent (low or insufficient adherence). Divergent results using the same scale have already been reported by other studies [35–37], which is to be expected since different populations were assessed. Nevertheless, no significant associations were observed between the CEAT-HIV adherence raw score and the direct measures (viral load and CD4 + count) in our study. While self-reported adherence has demonstrated positive correlation with viral load suppression in general populations without specificities, and is particularly suitable for resource-limited settings due to its low cost [33, 38], our divergent finding suggests the importance of assessing adherence based on both direct and indirect measures, especially in samples with specificities like ours, despite the potential limitations of a small sample size and reduced statistical power in the analysis.

However, despite the absence of an association between the CEAT-HIV score and viral load and CD4 + cell counts, the application of a self-reported adherence instrument in our context enabled the identification of factors negatively influencing medication adherence. These factors are amenable to intervention, given that the success of treatment depends on its continuity. It’s important to note that persistence in medication involves multiple factors, extending beyond those associated solely with the patient.

Access to information about one’s own condition and therapy is crucial for adherence to ART. Addressing topics such as what HIV and AIDS are, the asymptomatic and symptomatic stages, how the virus affects the body, the nature of therapy, and its potential side effects is essential for PLHIV to understand that the condition is chronic, requires continuous treatment, and should not be abandoned even in the absence of symptoms or when the patient feels well. Therefore, strengthening the doctor-patient relationship and helping the patient understand their condition emerges as a strategy to enhance therapy adherence [39]. 

Satisfaction with the treatment is a crucial factor for adherence, as well as for the perception of benefits associated with medication use and for strengthening the bond between the patient and health services [40]. The positive perception of clinical results achieved through adherence, coupled with strong relationships with health professionals and favorable treatment outcomes, likely contributed to increased satisfaction among study participants. This held true despite the high frequency of intense and moderate adverse effects observed in our data.

Factors such as care provided by a multidisciplinary team, home visits by therapeutic teams, shorter intervals between specialist consultations, proximity of the consultation location, satisfaction with care, and a positive relationship between professionals and users are crucial for enhancing adherence to ART. These findings confirm the pivotal role of health professionals in the therapeutic success of these patients [41, 42]. Additionally, evidence suggests that psychoeducational programs and motivational interviewing are effective strategies to improve adherence in PLHIV, particularly those with mental disorders [43]. Also, interventions that enhance social support have been shown to mitigate barriers related to stigma and isolation, thereby improving adherence outcomes [44, 45]. Incorporating these approaches into integrated care models may strengthen adherence and treatment outcomes in this vulnerable population.

This study has limitations, including reliance on a self-reported adherence scale, subject to participant response, which may lead to either overestimation or underestimation of the adherence rate. Due to the COVID-19 pandemic, interviews were conducted by telephone. However, we acknowledge that this method may have limited the depth of responses and impacted the accuracy of self-reported adherence. Other major limitation was the difficulty in contacting participants, largely due to outdated contact information, which reduced the number of individuals available for inclusion. Additionally, the clinical-epidemiological profile of the subjects was derived from computerized systems, and some information was entirely unavailable for certain individuals. As these data points did not exist in the database, they could not be considered in the analysis. However, variables with partially missing data were included in the analysis, with the extent of missing data being inferable from the frequency distribution tables. Finally, there is the limitation regarding the small number of individuals included in the study, and its specific setting in Campo Grande, Brazil, which may affect the generalizability of the findings.

Future research should aim to include larger and more heterogeneous samples across different regions and healthcare settings to enhance the external validity of findings and to better understand the factors influencing adherence in this population. In addition, employing mixed methods (combining self-reported adherence measures with objective data – such as pill counts, electronic monitoring, or pharmacy refill records) may offer a more comprehensive and accurate evaluation of adherence behaviors, while reducing the risk of bias inherent in self-report methods.

The findings of this study also underscore the need for healthcare policies that promote integrated care models, combining HIV treatment with mental health services. Strengthening psychosocial support and ensuring continuity of care for individuals with co-occurring HIV/AIDS and mental disorders are critical steps to improving adherence and health outcomes. Furthermore, policy initiatives should prioritize training healthcare professionals in mental health care and reducing barriers to access, particularly in resource-limited settings.

## Data Availability

No datasets were generated or analysed during the current study.

## References

[1] UNAIDS. Joint United Nations Programme on HIV/AIDS, Global HIV. & AIDS statistics — Fact sheet 2023. Available from: https://www.unaids.org/en/resources/fact-sheet. [Last accessed: October 1, 2023].

[2] Assefa Y, Gilks CF. Ending the epidemic of HIV/AIDS by 2030: Will there be an endgame to HIV, or an endemic HIV requiring an integrated health systems response in many countries? Int J Infect Dis. 2020; 100:273–277; 10.1016/j.ijid.2020.09.011 PMID: 32920236. 10.1016/j.ijid.2020.09.011 32920236

[3] World Health Organization. Global health sector strategies on, respectively, HIV, viral hepatitis and sexually transmitted infections for the period 2022–2030. 2022.

[4] Medley GF, Vassall A. When an emerging disease becomes endemic. Science. 2017;158(July):156–8. doi: 10.1126/science. aam8333 PMID: 28706039. 10.1126/science.aam8333 28706039

[5] Rocha GM, Machado CJ, Acurcio FA, et al. Monitoring adherence to antiretroviral treatment in brazil: an urgent challenge. Public Health J. 2011;27(SUPPL 1):67–78. 10.1590/s0102-311x2011001300008 21503526

[6] Lai HH, Wang CC, Yen TF, et al. Antiretroviral treatment adherence among people living with HIV in taipei, Taiwan. J Epidemiol Glob Health. 2024;14:1701–10. 10.1007/s44197-024-00329-y 39585641 10.1007/s44197-024-00329-y PMC11652450

[7] Nienke L, Elizabeth HG, Peter R, et al. Predictors and correlates of adherence to combination antiretroviral therapy (ART) for chronic HIV infection: a meta-analysis. BMC Med. 2014;12(1):1–14. 10.1186/s12916-014-0142-1 PMC4148019 25145556

[8] Nyaku M, Beer L, Shu F. Non-persistence to antiretroviral therapy among adults receiving HIV medical care in the united States. AIDS Care - Psychol Socio-Medical Asp AIDS/HIV. 2019;31(5):599–608. 10.1080/09540121.2018.1533232 10.1080/09540121.2018.1533232 30309269

[9] Parro-Torres C, Hernández-Huerta D, Ochoa-Mangado E et al. Antiretroviral treatment adherence and mental disorders: observational case-control study in people living with HIV in Spain. AIDS Care - Psychol Socio-Medical Asp AIDS/HIV. 2022; 34(8):1064–1072; 10.1080/09540121.2021.1944598 PMID: 34165358. 10.1080/09540121.2021.1944598 34165358

[10] Sued O, Rodriguez VJ, Weiss SM, et al. Antiretroviral therapy adherence and clinic attendance over time among people in Argentina living with HIV and lost to care. Int J Behav Med. 2025. 10.1007/s12529-025-10356-z 39994143 10.1007/s12529-025-10356-z PMC13342315

[11] Oh KS, Lee JS, Kim HC, Kang H-Y, Lee J-Y, Han E. Effects of depression on medication adherence in HIV/AIDS patients: Korea HIV/AIDS cohort study. J Infect Public Health. 2023;16:1598–605. 10.1016/j.jiph.2023.07.018 37573850 10.1016/j.jiph.2023.07.018

[12] Laranjeira C, Carvalho D, Valentim O, et al. Therapeutic adherence of people with mental disorders: an evolutionary concept analysis. Int J Environ Res Public Health. 2023;20(5). 10.3390/ijerph20053869. PMID: 36900879 PMCID: PMC10001153. 10.3390/ijerph20053869 PMC10001153 36900879

[13] Mandlate FM, Greene MC, Pereira LF, et al. Association between mental disorders and adherence to antiretroviral treatment in health facilities in two Mozambican provinces in 2018: a cross-sectional study. BMC Psychiatry. 2023;23:274. 10.1186/s12888-023-04782-0 37081470 10.1186/s12888-023-04782-0 PMC10116733

[14] Smith R, Villanueva G, Probyn K, Sguassero Y, Ford N, Orrell C, Cohen K, Chaplin M, Leeflang MMG, Hine P. Accuracy of measures for antiretroviral adherence in people living with HIV. Cochrane Database of Systematic Reviews 2022, Issue 7. Art. No.: CD013080; 10.1002/14651858.CD013080.pub2 10.1002/14651858.CD013080.pub2 PMC9309033 35871531

[15] Remor E, Milner-Moskovics J, Preussler G. Brazilian adaptation of the assessment of adherence to antiretroviral therapy questionnaire. Public Health J. 2007;41:685–94. 10.1590/s0034-89102006005000043 17713708

[16] de Brito BS, de Brito A, Monteiro EP, et al. Evidence of validity for the online version of the assessment of adherence to antiretroviral therapy questionnaire. SAGE Open. 2019;9(3):2158244019877201.

[17] Arashiro P, Maciel CG, Freitas FPR, et al. Adherence to antiretroviral therapy in people living with HIV with moderate or severe mental disorder. Sci Rep. 2023;13(1):3569. 10.1038/s41598-023-30451-z. PMID: 36864110 PMCID: PMC9980869. 36864110 10.1038/s41598-023-30451-z PMC9980869

[18] Eggers C, Arendt G, Hahn K, et al. HIV-1-associated neurocognitive disorder: epidemiology, pathogenesis, diagnosis, and treatment. J Neurol. 2017;264:1715–27. 28567537 10.1007/s00415-017-8503-2 PMC5533849

[19] Saylor D, Dickens AM, Sacktor N, et al. HIV-associated neurocognitive disorder—pathogenesis and prospects for treatment. Nat Reviews Neurol. 2016;12(4):234–48. 10.1038/nrneurol.2016.27 PMC4937456 26965674

[20] Carvalho PMM, Silva Neto RM, Rolim Neto ML. Fatores de Saúde mental associados à Não Adesão à terapia anti-retroviral: uma Revisão Sistemática. Revista Saúde Coletiva. 2020;10:3665–77.

[21] Zuge SS, Brum CN, João Vitor V et al. Associação entre Adesão à Medicação para o HIV e Aspectos Sociodemográficos, Econômicos, Comportamentais e Clínicos. Revista Contexto & Saúde, v. 21, n. 44, out./dez. 2021. 10.21527/2176-7114.2021.44.11919

[22] Ministério da Saúde. Boletim Epidemiológico HIV / AIDS Boletim Epidemiológico HIV/AIDS. 2022. Available from: https://www.gov.br/aids/pt-br/central-de-conteudo/boletins-epidemiologicos/2022/hiv-aids/boletim_hiv_aids_-2022_internet_31-01-23.pdf/view. [Last accessed: September 22, 2023].

[23] Grangeiro A, Escuder MM, Cassanote AJF et al. The HIV-Brazil cohort study: design, methods and participant characteristics. PLoS ONE 2014;9(5):e95673. 10.1371/journal.pone.0095673 PMC4006775 24789106

[24] Smith AB, Cook PF. Comorbid mental health disorders in persons living with HIV: Adherence to antiretroviral therapy. Archives of Psychiatric Nursing. 2019; v. 33, n. 4, pp. 364–370. 10.1016/j.apnu.2019.04.008. PMID: 31280781; PMCID: PMC6814186. 10.1016/j.apnu.2019.04.008 PMC6814186 31280781

[25] Ma J, Luu B, Ruderman SA, Whitney BM, Merrill JO, Mixson LS, Nance RM, et al. Alcohol and drug use severity are independently associated with antiretroviral adherence in the current treatment era. AIDS Care. 2023;36(5):618–30. 10.1080/09540121.2023.2223899 37419138 10.1080/09540121.2023.2223899 PMC10771542

[26] Olashore AA, Chiliza B, Paruk S. Antiretroviral therapy non-adherence and its relationship with cognitive impairment, alcohol use disorder, and depression in adolescents living with HIV. BMC Psychiatry. 2023;23(1):532. 10.1186/s12888-023-05000-7 37488527 10.1186/s12888-023-05000-7 PMC10367307

[27] Gizachew KD, Chekol YA, Basha EA, et al. Suicidal ideation and attempt among people living with HIV/AIDS in selected public hospitals: central Ethiopia. Ann Gen Psychiatry. 2021;20(1):15. 10.1186/s12991-021-00335-5 33608017 10.1186/s12991-021-00335-5 PMC7896396

[28] Nguyen MX, McNaughton RHL, Pence BW et al. The longitudinal association between depression, anxiety symptoms and HIV outcomes, and the modifying effect of alcohol dependence among ART clients with hazardous alcohol use in Vietnam. J Int AIDS Soc. 2021;24:e25746. 10.1002/jia2.25746 PMC8222856 34165258

[29] Wagner GJ, Kanouse DE, Koegel P, et al. Adherence to HIV antiretrovirals among persons with serious mental illness. AIDS Patient Care STDs. 2003;17(4):179–86. 12737641 10.1089/108729103321619782

[30] Remien RH, Stirratt MJ, Nguyen N, et al. Mental health and HIV/AIDS: the need for an integrated response. AIDS. 2019;33(9):1411. 30950883 10.1097/QAD.0000000000002227 PMC6635049

[31] Rocha GM, Machado CJ, Acurcio FDA, et al. Monitoring adherence to antiretroviral treatment in brazil: an urgent challenge. Public Health J. 2011;27:s67–78. 10.1590/s0102-311x2011001300008 21503526

[32] Costa JO, Schaffer AL, Medland NA et al. Adherence to antiretroviral regimens in Australia: A nationwide cohort study. AIDS Patient Care STDs. 2020. 34, 81–91. 10.1089/apc.2019.0278 PMID: 32049558. 10.1089/apc.2019.0278 32049558

[33] Bezabhe WM, Peterson GM, Bereznicki L et al. Adherence to antiretroviral drug therapy in adult patients who are HIV-positive in Northwest ethiopia: a study protocol. BMJ Open 2013;3(10):e003559. 10.1136/bmjopen-2013-003559 PMC3816234 24176794

[34] Shushtari ZJ, Salimi Y, Sajjadi H, Paykani T. Effect of social support interventions on adherence to antiretroviral therapy among people living with HIV: A systematic review and meta-analysis. AIDS Behav. 2023;27(5):1619–35. 36318421 10.1007/s10461-022-03894-0

[35] Biney IJK, Kyei KA, Ganu VJ, et al. Antiretroviral therapy adherence and viral suppression among HIV-infected adolescents and young adults at a tertiary hospital in Ghana. Afr J AIDS Res. 2021;20(4):270–6. 34905452 10.2989/16085906.2021.1998783

[36] Menezes EG, Santos SRFD, Melo GZDS, et al. Fatores associados à Não Adesão Dos antirretrovirais Em portadores de HIV/AIDS. Acta Paulista De Enfermagem. 2018;31:299–304.

[37] Miranda MDMF, Oliveira DRD, Quirino GDS et al. Adherence to antiretroviral therapy among adults living with HIV/AIDS: a cross-sectional study. Brazilian Nurs J 2022;75(2):e20210019. 10.1590/0034-7167-2021-0019 10.1590/0034-7167-2021-0019 34669905

[38] McMahon JH, Jordan MR, Kelley K, et al. Pharmacy adherence measures to assess adherence to antiretroviral therapy: review of the literature and implications for treatment monitoring. Clin Infect Dis. 2011;52(4):493–506. 21245156 10.1093/cid/ciq167 PMC3060901

[39] Brasil. Ministério da Saúde. Protocolo Clínico e Diretrizes Terapêuticas para Manejo da Infecção pelo HIV em Adultos. Brasília: MS; 2023. Available from: https://www.gov.br/aids/pt-br/central-de-conteudo/pcdts/2013/hiv-aids/pcdt_manejo_adulto_12_2018_web.pdf. [Last accessed: September 1, 2023].

[40] Koga I, Wakatabe R, Okamoto N, et al. Factors associated with treatment satisfaction among people living with HIV in Japan and other selected countries: examination of the intertwined roles of medication, patient, and provider characteristics. AIDS Behav. 2022;26(5):1633–51. 10.1007/s10461-021-03515-2] 34870772 10.1007/s10461-021-03515-2 PMC8647062

[41] Bonolo P, Gomes RRFM, Guimarães MD. Adherence to antiretroviral therapy (HIV/AIDS): associated factors and adherence measures. Epidemiol Health Serv. 2007;16(4):267–78.

[42] Carvalho PP, Barroso SM, Coelho HC et al. Fatores associados à adesão à Terapia Antirretroviral em adultos: revisão integrativa de literatura. Ciência & Saúde Coletiva, 2019. v. 24, n. 7, pp. 2543-55. Available from: https://www.scielo.br/j/csc/a/hwgHkxJgkv5TpcVPVTtsLxs/. [Last accessed: September 22, 2023]. 10.1590/1413-81232018247.22312017 31340272

[43] Papus M, Dima AL, Viprey M, Schott AM, Schneider MP, Novais T. Motivational interviewing to support medication adherence in adults with chronic conditions: systematic review of randomized controlled trials. Patient Educ Couns. 2022;105(11):3186–203. 10.1016/j.pec.2022.06.013 35779984 10.1016/j.pec.2022.06.013

[44] Mireles L, Horvath KJ, Guadamuz TE, et al. The moderating role of social support and HIV stigma on the association between depression and ART adherence among young Thai men who have sex with men. AIDS Behav. 2023;27:2959–68. 10.1007/s10461-023-04018-y 37000384 10.1007/s10461-023-04018-y PMC10524997

[45] Ajuna N, Tumusiime B, Amanya J, Awori S, Rukundo GZ, Asiimwe JB. (2021). Social Networks and Barriers to ART Adherence Among Young Adults (18–24 years) Living with HIV at Selected Primary Health Facilities of South-Western Uganda: A Qualitative Study. HIV/AIDS - Research and Palliative Care, 13, 939–958; 10.2147/HIV.S328643 10.2147/HIV.S328643 PMC8504700 34675686

